# Association between body mass index and disability among older population in China: analysis of 2011–2020 data from the China Health and Retirement Longitudinal Study

**DOI:** 10.7189/jogh.15.04277

**Published:** 2025-12-05

**Authors:** Rongrong Guo, Shan Zhang, Ting Fu, Yushan Guan, Yuan Luo, Ying Wu

**Affiliations:** 1 School of Nursing, Capital Medical University, Beijing, China; 2 Nursing Department, Affiliated Hospital of Nantong University, Nantong, China; 3 Chinese Institutes for Medical Research, Beijing, China

## Abstract

**Background:**

The study explored the relationship between body mass index (BMI) and activities of daily living (ADL) disability among Chinese older adults.

**Methods:**

Using 2011–2020 data from the China Health and Retirement Longitudinal Study, we included 3975 older individuals and assessed their baseline BMI, ADL disability, other covariates, and ADL disability over the follow-up period. Cox proportional hazards regression, restricted cubic spline, and two-piecewise linear regression models were performed. We also conducted subgroup analyses to explore effect heterogeneity across different subpopulations and sensitivity analyses to confirm the robustness of our findings.

**Results:**

During a median follow-up of seven years, 2003 participants developed ADL disability. The Cox proportional hazards models demonstrated a significant association between BMI and the risk of ADL disability. When BMI was categorised into groups, only obese older adults exhibited a significantly higher risk of ADL disability compared to those with normal weight. The restricted cubic spline model further revealed a nonlinear U-shaped relationship between continuous BMI and ADL disability risk, indicating that the risk of ADL disability initially decreased and then increased with rising BMI. Subgroup analyses revealed that the U-shaped relationship was observed only among individuals aged 60–69 years and female older adults, while sensitivity analyses consistently confirmed the robustness of this U-shaped association between BMI and ADL disability risk.

**Conclusions:**

A nonlinear U-shaped relationship between BMI and ADL disability risk was observed among Chinese adults aged 60–69 and older female adults, suggesting that both high and low BMI are associated with increased ADL disability risk. Despite limitations such as baseline-only BMI measurements, observational study design, potential residual confounding, and limited generalisability beyond Chinese older adults, these findings highlight the importance of routine BMI screening and targeted weight management strategies to help prevent or delay the onset of ADL disability in older adults.

With the development of population aging and rising chronic disease prevalence, a growing number of older adults are currently and projected to live with disabilities [1]. In China, approximately 52.7 million older adults were living with disabilities in 2020, and this number is expected to surge to 95.4 million by 2050 [2]. International Classification of Functioning, Disability and Health defined disability as either intrinsic to the individual or resulting from the interaction between the individual’s physical and social environment [3], with activities of daily living (ADL) disability as one of the most severe types [4]. Considered as an irreversible condition with difficulty in undertaking activities in any area of daily life, ADL disability is linked to future falls, dependency, cognitive decline, decreased life satisfaction, and even mortality [5]. Therefore, to prevent and postpone ADL disability in older adults, accurate identification of risk factors is essential to select high-risk individuals and thus implement early interventions.

Body mass index (BMI), a simple and widely used indicator of body fat [6], has been reported to show a U-shaped relationship with ADL disability in adults, where both low and high BMI increase ADL disability risk [4]. However, age-related changes in body composition, such as fat redistribution, increased visceral and intramuscular fat, sarcopenia, and bone marrow fat, make older adults more vulnerable to the adverse effects of abnormal BMI, regardless of weight stability [7]. These physiological changes complicate the relationship between BMI and ADL disability risk among older adults, the age group most susceptible to ADL disability [8].

Although studies in high-income countries such as America [9], Japan [10], and New Zealand [11] consistently reported a U-shaped association between BMI and ADL disability risk among older adults, findings from China, a middle-income country with the world’s largest older population, have been varied [12]. This divergence may stem from China’s unique socio-demographic context, including rapid urbanisation, nutritional transitions, and pronounced rural–urban disparities in health care access. First, rapid urbanisation and internal migration have weakened traditional family-based care, especially in rural areas, increasing vulnerability to functional decline. Second, shifting from undernutrition to overnutrition has led to a double burden of malnutrition and obesity. Third, limited access to preventive care, rehabilitation, and chronic disease management in rural areas may further influence the BMI–ADL disability relationship.

In addition to these cross-national differences, evidence within China is inconsistent. A cross-sectional study using 2006 China Health and Nutrition Surveys data found no significant association among adults aged 55 and older [13]. In contrast, another cross-sectional study of nonagenarians and centenarians reported a U-shaped association only in females but not in males [14]. Similarly, a cross-sectional study based on the Chinese Longitudinal Healthy Longevity Study supported a J-shaped and inverse J-shaped relationship among males and females aged 80 and above, indicating that higher BMI was associated with increased ADL disability risk in males but decreased risk in females [15]. Meanwhile, a longitudinal study based on the Chinese Longitudinal Healthy Longevity Study data found a linear association between BMI and ADL disability risk, but this study exclusively included the oldest-old (80+), leaving younger older adults (aged 60–80) underrepresented [4]. Potential explanations for such inconsistencies include differences in study design, participant age ranges, definitions of ADL disability, and unmeasured contextual factors such as diet, physical activity levels, or urban-rural residence status.

Therefore, the current study aimed to explore the longitudinal association between BMI and ADL disability risk among Chinese populations aged 60 and above by using data from the China Health and Retirement Longitudinal Study, a nationally representative cohort. Specifically, we conducted subgroup analyses by age group, gender, and other key demographic factors to identify variations across population groups. These findings may provide valuable evidence for identifying vulnerable groups and developing targeted strategies to prevent or postpone ADL disability in Chinese older population.

## METHODS

### Study design and participants

We used data the China Health and Retirement Longitudinal Study did in 2011, 2013, 2015, 2018, and 2020. The detailed study design has been published previously [16] and was briefly described in Method S1 in the **Online Supplementary Document**.

In the current study, we included individuals aged 60 years or older at baseline with at least one follow-up data. Due to the absence of BMI data in 2018 and the lack of follow-up data for participants enrolled in 2020, we included participants enrolled in 2011, 2013, and 2015. We excluded individuals with missing data on age, BMI, and ADL disability, as well as those identified with ADL disability at their initial assessment. Additionally, participants without follow-up data on ADL disability were excluded. Written informed consent was obtained from participants or their legal representatives at both baseline and follow-up assessments. The current study was reported following the Strengthening the Reporting of Observational Studies in Epidemiology reporting guideline [17].

### Ascertainment of ADL disability

We defined ADL disability as the inability to independently perform any basic ADL, including feeding, dressing, moving from bed to chair, using the toilet, bathing, and maintaining continence. Participants were recorded as ADL disability ‘yes’ as long as one item of ADLs was recorded as ‘have difficulty and need help’, ‘with difficulty but still can do it’ or ‘cannot do it’, or otherwise ‘no’ [18].

### Measurement and calculation of BMI

Height and weight measured at baseline were used to calculate BMI. Weight was measured in light clothes without shoes to the nearest 0.1 kg on an OmronTM HN-286 scale. Height was measured without shoes to the nearest 0.1 cm using a SecaTM 213 m. We calculated BMI as weight (kg) divided by squared height (m^2^) and rounded to two decimal places. According to Chinese obesity guideline [19], BMI was categorised as underweight (<18.5), normal weight (18.5–23.9), overweight (24.0–27.9), and obesity (≥28.0).

### Assessment of covariates

Potential covariates were selected based on the Disablement Process Model by Verbrugge and Jette [20], which encompasses sociodemographic characteristics, diseases, functional impairments, psychological and cognitive status, health behaviours, and social support. Confounders of the BMI–ADL disability association were identified from prior systematic reviews and exploratory studies. Details on covariate items, definitions, and coding were provided in Method S2 and Table S1 in the **Online Supplementary Document**.

### Statistical analyses

To minimise potential bias and preserve statistical power, we employed Multiple Imputation by Chained Equations to generate 10 complete data sets under the assumption that data were missing at random [21]. Each data set was analysed separately and results were pooled using the ‘with’ function of the ‘mice’ package.

Continuous variables were presented as either means ± standard deviation or medians (25% percentile, 75% percentile) as appropriate, and categorical variables were expressed as frequencies and proportions. Kruskal-Wallis H test or χ^2^ test was performed to compare continuous or categorical variables across BMI categories. Association between BMI and ADL disability risk was analysed using a multivariate Cox proportional hazards regression model to estimate hazard ratios and 95% confidence intervals. Proportional hazard assumption was examined using Schoenfeld residuals. Unadjusted Model 1 was developed first, and sociodemographic characteristics were adjusted in Model 2, including age, gender, education, marital status, residence area, and annual *per capita* household expenditure. We further adjusted for diseases in Model 3, including hypertension, diabetes or high blood glucose, stroke, cancer, heart problems, chronic lung diseases, and arthritis. For Model 4, we additionally adjusted for smoking, alcohol drinking, low grip strength, vision impairment, hearing impairment, cognitive decline, depression, monthly social engagement, Short Physical Performance Battery level, and fall history. Multicollinearity was assessed by variation inflation factors (VIFs), with VIFs between 1–4 indicating no multicollinearity. Model performance was assessed using the Akaike Information Criterion (AIC) and concordance index, with lower AIC and higher concordance index suggesting better fit and discrimination. A restricted cubic spline (RCS) model was performed to visually show the dose-response relationship between continuous BMI and ADL disability, with knots (4–7) selected based on the lowest AIC [22]. If a nonlinear association was identified, a two-piecewise linear regression model was applied, using the inflection point as the threshold for stratified analysis.

To determine whether demographic characteristics modified the association, we conducted subgroup analyses by age group (60–69, 70–79, 80+), gender, marital status, education, and residence area. Interactions between stratification factors and BMI were examined by including two-factor interaction terms in the Cox regression model.

Several sensitivity analyses were conducted to evaluate the robustness of our findings. First, to reduce reverse causality, we performed a lagged panel analysis and excluded participants who died during follow-up. Second, to account for BMI fluctuations over time, we restricted analyses to participants with stable BMI (BMI at the second assessment differed from baseline by ≤5%) and used BMI measured at the second wave for model construction. Third, to address potential baseline misclassification and early-onset disability, we excluded individuals who developed ADL disability at the first follow-up. Fourth, we repeated analyses in individuals without missing covariates. Fifth, given that cognitive decline, depression, low grip strength, hypertension, diabetes or high blood glucose, stroke, heart diseases, cancer, arthritis, fall history, hearing impairment, and vision impairment may act as confounding factors in the association between BMI and ADL disability risk [23], we repeated analyses by sequentially excluding participants with each of these conditions to minimise potential confounding effects.

Data were cleaned using STATA SE 15 (StataCorp, College Station, Texas, USA) and all analyses were performed using R Studio 4.2.0 (RStudio, Boston, MA, USA). A two-sided *P* < 0.05 was considered statistically significant.

## RESULTS

### Baseline characteristics of the study participants

**Figure 1:**
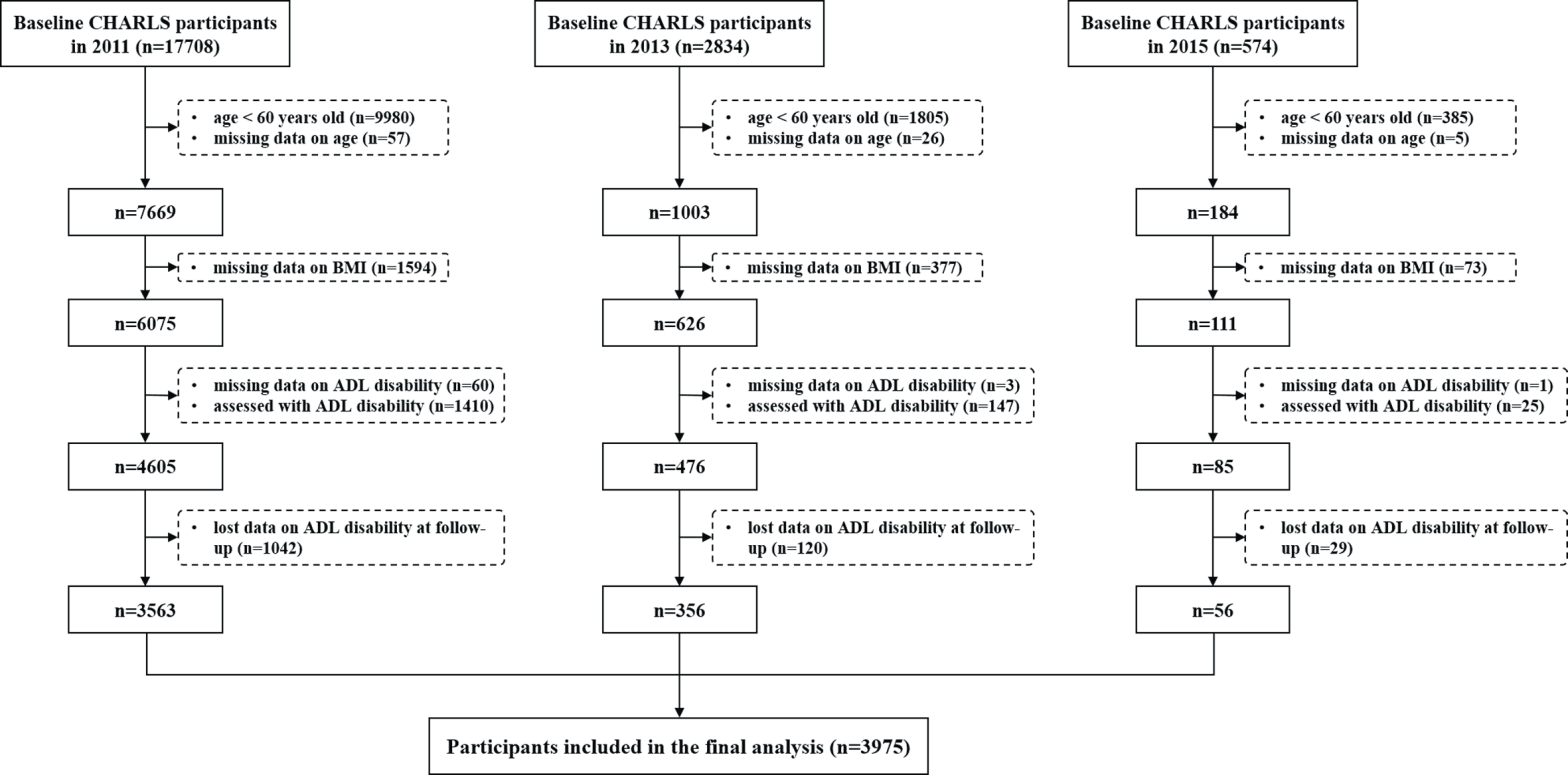
Flowchart of the included study participants.

The inclusion process was presented in **Figure 1**. A total of 3975 older individuals were included. After processing missing data (Table S2 in the **Online Supplementary Document**), participants’ baseline characteristics were summarised in **Table 1**. The median age of participants was 66 (interquartile range (IQR) = 62.00–71.00) with 50.94% being male. The median BMI was 22.53 (IQR = 20.26–25.11) with 9.43, 55.50, 25.79, and 9.28% classified as underweight, normal weight, overweight, and obese, respectively. Older adults with higher BMI tended to be younger, female, urban residents, with lower rates of hearing impairment and higher prevalence of hypertension, diabetes or high blood glucose, heart diseases, and stroke. Over a median follow-up of 7.00 years (IQR = 4.00–9.00), 2003 participants developed ADL disability. The incidence of ADL disability was 54.93% in the underweight group, 48.64% in normal weight, 49.37% in overweight, and 59.08% in obesity.

**Table 1:** Baseline characteristics of the study participants stratified by BMI categories

Variables	Overall (n = 3975)	Underweight (n = 375)	Normal weight (n = 2206)	Overweight (n = 1025)	Obesity (n = 369)	*P*_trend_
Age	66.00 (62.00–71.00)	69.00 (64.00–74.75)	66.00 (62.00–71.00)	65.00 (62.00–69.00)	64.00 (62.00–69.00)	<0.001*
Male	2025 (50.94%)	180 (48.00%)	1245 (56.44%)	464 (45.27%)	136 (36.87%)	<0.001*
BMI (kg/m^2^)	22.53 (20.26–25.11)	17.52 (16.73–18.04)	21.36 (20.10–22.64)	25.48 (24.69–26.55)	29.66 (28.74–31.21)	<0.001*
Marital status						<0.001*
*Married*	3225 (81.13%)	278 (74.13%)	1771 (80.28%)	873 (85.17%)	303 (82.11%)	
*Unmarried*	750 (18.87%)	97 (25.87%)	435 (19.72%)	152 (14.83%)	66 (17.89%)	
Education						<0.001*
*Illiterate*	1409 (35.45%)	172 (45.87%)	778 (35.27%)	325 (31.71%)	134 (36.31%)	
*Primary or below*	1848 (46.49%)	167 (44.53%)	1047 (47.46%)	471 (45.95%)	163 (44.17%)	
*Secondary or above*	718 (18.06%)	36 (9.60%)	381 (17.27%)	229 (22.34%)	72 (19.51%)	
Urban area	1429 (35.95%)	99 (26.40%)	678 (30.73%)	454 (44.29%)	198 (53.66%)	<0.001*
Annual *per-capita* household expenditure						<0.001*
*Low level*	2652 (66.72%)	285 (76.00%)	1546 (70.08%)	623 (60.78%)	198 (53.66%)	
*Middle level*	906 (22.79%)	70 (18.67%)	456 (20.67%)	274 (26.73%)	106 (28.73%)	
*High level*	417 (10.49%)	20 (5.33%)	204 (9.25%)	128 (12.49%)	65 (17.62%)	
Grip strength (kg)	27.80 (21.50–35.00)	23.75 (19.00–30.38)	28.00 (21.50–35.00)	28.50 (22.50–36.13)	28.00 (22.50–35.50)	<0.001*
Smoking						<0.001*
*Never smoker*	2273 (57.18%)	201 (53.60%)	1165 (52.81%)	649 (63.32%)	258 (69.92%)	
*Former smoker*	434 (10.92%)	29 (7.73%)	229 (10.38%)	124 (12.10%)	52 (14.09%)	
*Current smoker*	1268 (31.90%)	145 (38.67%)	812 (36.81%)	252 (24.59%)	59 (15.99%)	
Alcohol drinking						<0.001*
*Never drinker*	2309 (58.09%)	241 (64.27%)	1207 (54.71%)	623 (60.78%)	238 (64.50%)	
*Former drinker*	422 (10.62%)	34 (9.07%)	251 (11.38%)	95 (9.27%)	42 (11.38%)	
*Current drinker*	1244 (31.30%)	100 (26.67%)	748 (33.91%)	307 (29.51%)	89 (24.12%)	
Distant vision impairment	1071 (26.94%)	110 (29.33%)	605 (27.43%)	261 (25.46%)	95 (25.75%)	0.431
Near vision impairment	915 (23.02%)	91 (24.27%)	492 (22.30%)	254 (24.78%)	78 (21.14%)	0.321
Hearing impairment	719 (18.09%)	87 (23.20%)	411(18.63%)	166 (16.20%)	55 (14.91%)	0.007*
Hypertension	1137 (28.60%)	50 (22.71%)	501 (13.33%)	387 (37.76%)	199 (53.93%)	<0.001*
Heart problems	552 (13.89%)	36 (9.60%)	261 (11.83%)	164 (16.00%)	91 (24.66%)	<0.001*
Diabetes or high blood glucose	258 (6.49%)	10 (2.67%)	98 (4.44%)	101 (9.85%)	49 (13.28%)	<0.001*
Chronic lung diseases	510 (12.83%)	73 (19.47%)	290 (13.15%)	108 (10.54%)	39 (10.57%)	<0.001*
Cancer	44 (1.11%)	6 (1.60%)	23 (1.04%)	11 (1.07%)	4 (1.08%)	0.819
Stroke	87 (2.19%)	7 (1.87%)	36 (1.63%)	29 (2.83%)	15 (4.07%)	0.010*
Arthritis	1339 (33.69%)	121 (32.27%)	738 (33.45%)	340 (33.17%)	140 (37.94%)	0.320
Monthly social engagement	1900 (47.80%)	159 (42.40%)	1008 (45.69%)	532 (51.90%)	201 (54.47%)	<0.001*
Cognitive Decline	577 (14.52%)	90 (24.00%)	344 (15.59%)	106 (10.34%)	37 (10.03%)	<0.001*
Depression	1137 (28.60%)	148 (39.47%)	647 (29.33%)	254 (24.78%)	88 (23.85%)	<0.001*
Low grip strength	687 (17.28%)	106 (28.67%)	414 (28.27%)	121 (11.80%)	46 (12.47%)	<0.001*
SPPB level						<0.001*
*Low level*	283 (7.12%)	30 (8.00%)	147 (6.66%)	70 (6.83%)	36 (9.76%)	
*Middle level*	1280 (32.20%)	151 (40.27%)	693 (31.41%)	304 (29.66%)	132 (35.77%)	
*High level*	2412 (60.68%)	194 (51.73%)	1366 (61.92%)	651 (63.51%)	201 (54.47%)	

BMI – body mass index, SPPB – Short Physical Performance Battery

*Statistically significant (*P*_trend_<0.05).

### Association between BMI and ADL disability

**Figure 2:**
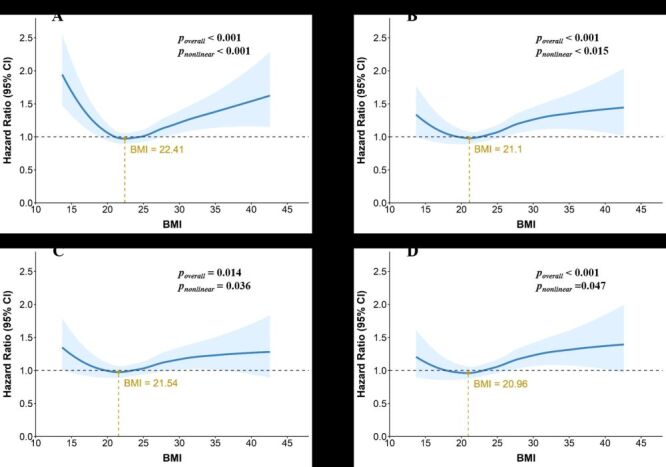
Restricted cubic spline curves between continuous BMI and ADL disability. **Panel A.** For Model 1. **Panel B.** For Model 2. **Panel C.** For Model 3. **Panel D.** For Model 4. ADL – activities of daily living, BMI – body mass index.

Cox regression results were presented in **Table 2**. Continuous BMI was positively associated with ADL disability risk in the unadjusted Model 1 (**Figure 2**, Panel A), and this association remained significant across Model 2 (**Figure 2**, Panel B), Models 3 (**Figure 2**, Panel C), and Model 4 (**Figure 2**, Panel D) after stepwise covariate adjustment, indicating a consistent association between higher BMI and an elevated ADL disability risk. In categorical analyses using normal weight as the reference, both underweight and obesity were associated with higher risks of ADL disability in Model 1. However, after covariate adjustment, the association for underweight was no longer significant, while the association for the obesity group remained robust. Model 4 demonstrated the best fit, with the lowest AIC value and highest concordance index (Table S3 in the **Online Supplementary Document**). Additionally, all covariates had VIFs between 1–4, suggesting no multicollinearity.

**Table 2:** Cox regression model results for the association between BMI and ADL disability

BMI	No. of events	Model 1*	Model 2†	Model 3‡	Model 4§
		**HR (95% CI), *P*-value¶**	**HR (95% CI), *P*-value¶**	**HR (95% CI), *P*-value¶**	**HR (95% CI), *P*-value¶**
Continuous					
*Per increase*	2003/3975	1.013 (1.002–1.025); *P* = 0.021	1.019 (1.008–1.030); *P* < 0.001	1.012 (1.001–1.024); *P* = 0.038	1.020 (1.008–1.031); *P* < 0.001
Categories					
*Underweight*	206/375	1.267 (1.092–1.471); *P* = 0.002	1.080 (0.929–1.255); *P* = 0.318	1.079 (0.928–1.255); *P* = 0.322	1.023 (0.879–1.190); *P* = 0.774
*Normal weight*	1073/2206	Reference	Reference	Reference	Reference
*Overweight*	506/1025	1.027 (0.924–1.141); *P* = 0.622	1.088 (0.977–1.212); *P* = 0.124	1.042 (0.934–1.162); *P* = 0.463	1.077 (0.965–1.203); *P* = 0.186
*Obesity*	218/369	1.349 (1.166–1.560); *P* < 0.001	1.414 (1.218–1.641); *P* < 0.001	1.277 (1.096–1.488); *P* = 0.002	1.369 (1.173–1.598); *P* < 0.001

ADL – activities of daily living, BMI – body mass index

*Unadjusted.

†Adjusted for age, gender, education, marital status, residence area, and annual *per capita* household expenditure.

‡Adjusted for age, gender, education, marital status, residence area, annual *per capita* household expenditure, hypertension, diabetes or high blood glucose, stroke, cancer, heart problems, chronic lung diseases, and arthritis.

§Adjusted for age, gender, education, marital status, residence area, annual *per capita* household expenditure, hypertension, diabetes or high blood glucose, stroke, cancer, heart problems, chronic lung diseases, arthritis, smoking, drinking, low grip strength, vision impairment, hearing impairment, cognitive decline, depression, monthly social engagement, Short Physical Performance Battery level, and fall history.

¶Statistically significant (*P* < 0.05).

### Dose-response relationship between BMI and ADL disability

Based on the lowest AIC, six knots were used for the RCS model. As demonstrated in **Figure 2**, Model 1 revealed a significant U-shaped association between continuous BMI and ADL disability risk, with the lowest risk at 22.41 kg/m^2^. This pattern persisted after adjusting for confounders in Models 2–4, showing a decline in risk with increasing BMI up to the threshold, followed by a significant rise. Specific BMI cutoffs and corresponding hazard ratios were presented in Table S4 in the **Online Supplementary Document**.

### Subgroup analyses

**Figure 3:**
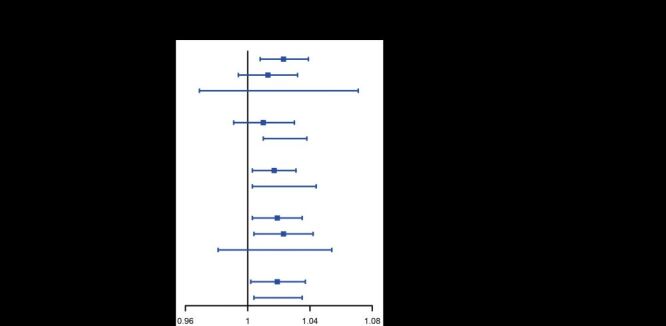
Subgroup analyses of associations between continuous BMI and ADL disability. All models were fully adjusted by age, gender, education, marital status, residence area, annual *per capita* household expenditure, hypertension, diabetes or high blood glucose, stroke, cancer, heart problems, chronic lung diseases, arthritis, smoking, drinking, low grip strength, vision impairment, hearing impairment, cognitive decline, depression, monthly social engagement, SPPB level, and fall history. ADL – activities of daily living, BMI – body mass index, SPPB – Short Physical Performance Battery.

Subgroup analysis results were summarised in **Figure 3**; Table S5 in the **Online Supplementary Document**. When stratified by age, a significant association between continuous BMI and ADL disability risk was observed only among individuals aged 60–69 years, with no association in older age groups. In gender-stratified analysis, the association remained significant in females but not in males. Regarding marital status, the association was significant in both married and unmarried individuals. By education level, the association was evident among those with no formal education and with primary education or below, but not among those with secondary education or above. Regarding residence area, the association was consistent across urban and rural residents. No significant interaction effects were detected (*P*_interaction_>0.05).

As shown in Figure S1–5 in the **Online Supplementary Document**, the RCS curves showed a persistent U-shaped relationship among adults aged 60–69, unmarried individuals, and rural residents after full adjustment. In contrast, a linear association was observed in females, married individuals, those with lower education, and urban residents.

### Sensitivity analyses

Sensitivity analyses showed no substantial changes in Cox regression results, supporting the robustness of our findings. Results from the RCS models also remained consistent, though slight linearity appeared after excluding participants with missing covariates, depression, arthritis, or hearing impairment. Detailed results were presented in Table S6–7 and Figure S6 in the **Online Supplementary Document**.

## DISCUSSION

In this nationally representative longitudinal study, we found a nonlinear U-shaped relationship between BMI and ADL disability risk among Chinese adults aged 60–69 years and female older adults, with both low and high BMI are associated with an increased risk of ADL disability. This finding fills an important knowledge gap. The existing studies in China reported inconsistent associations, whereas our results identified a U-shaped relationship consistent with findings from studies in high-income countries. To the best of our knowledge, this is the first large-scale cohort study examining the relationship between BMI and ADL disability in Chinese older adults.

Our Cox regression and RCS model results exhibit strong face validity for several key reasons. First, the observed 50.39% incidence of ADL disability aligns with previous studies, confirming our sample’s representativeness [18,24]. Second, the identified U-shaped relationship between BMI and ADL disability risk is consistent with several prior studies in China. For example, both a cross-sectional study in Dujiangyan [14] and a survey of community-dwelling older adults in Shanghai [25] reported significantly elevated risks of ADL disability among underweight and obese older adults, with these associations remaining significant after confounder adjustment. Moreover, our findings are supported by evidence from several international longitudinal studies. An investigation involving middle-aged and older adults in the USA demonstrated that both underweight and obesity were significantly associated with increased risks of ADL disability, although the association for underweight was non-significant in individuals aged 70–96 years [9]. Similarly, a Japanese cohort study identified a U-shaped association across sexes, with both underweight and obese older adults exhibiting markedly higher ADL disability risks [10]. Additionally, a large-scale cohort study in New Zealand confirmed the U-shaped association among nursing home residents [11].

Consistent with previous studies targeting older adults, an elevated BMI was found to be associated with increased ADL disability risk, which remained robust in the categorical analysis. Several reasons may explain this (Figure S7 in the **Online Supplementary Document**). First, elevated BMI increases mechanical load on weight-bearing joints such as the knees and hips, accelerating degenerative changes and causing joint pain, impaired balance, and gait instability [24]. These impairments contribute to physical inactivity, muscle atrophy, and bone density loss, ultimately promoting ADL disability [25]. Second, higher BMI is associated with metabolic dysregulation and chronic inflammation [26,27], both of which increase the risk of developing chronic diseases such as cardiovascular diseases, diabetes mellitus, and stroke [28,29]. These conditions reduce exercise tolerance and hinder independent performance of daily activities, thereby increasing the likelihood of ADL impairment [30]. Third, excessive visceral fat accumulation triggers low-grade inflammation characterised by elevated circulating pro-inflammatory cytokines [31]. These inflammatory mediators can inhibit muscle protein synthesis while activating protein degradation, leading to progressive muscle loss and, ultimately, ADL disability [32].

Although several studies suggested lower BMI may increase ADL disability risk, this association emerged only when BMI was treated as a continuous variable in our study. One possible explanation is that BMI does not differentiate between fat mass and lean mass, nor does it reflect fat distribution [33], which complicates the interpretation. Moreover, the relatively small sample size of the underweight group may limit the statistical power to detect a significant association. Residual confounding or reverse causality, such as underlying health conditions causing both low BMI and disability, may also have attenuated the observed associations [34]. Finally, categorising BMI could result in information loss, obscuring associations evident in continuous modelling. Further studies with larger underweight samples and more detailed body composition measures are needed to clarify the relationship between a decreased BMI and ADL disability risk in Chinese older individuals.

Interestingly, our age-stratified analysis showed a significant U-shaped association only among older individuals aged 60–69, with no such relationship found in those aged 70 and above. This age-dependent difference may reflect several factors. First, survival bias may play a crucial role, as individuals with higher BMI who survive into advanced age tend to represent a healthier subset, the so-called ‘healthy survivor’ effect, thereby attenuating or even eliminating the association between BMI and ADL disability. Second, adults aged 70 and above are more likely to experience physical function decline, sarcopenia, and the accumulation of multiple chronic conditions [35], which may obscure the direct relationship between BMI and ADL disability. Additionally, the relatively small sample sizes in the 70–79 and 80+ age groups may lack sufficient statistical power to draw robust conclusions.

In the gender-based subgroup analysis, a U-shaped relationship was found only in females, consistent with previous studies [14]. This may relate to variations in adipose tissue distribution, with older females being more prone to central obesity [36]. As obesity progresses, declining oestrogen levels in postmenopausal women may reduce their capacity to store fat in subcutaneous tissue and lead to muscle loss, thereby increasing their vulnerability to ADL disability [37,38]. The absence of a significant association in males could stem from limited sample size, and the non-significant gender-by-BMI interaction suggests inconclusive gender differences. Therefore, future studies should include larger sex-stratified cohorts and account for physiological factors, such as fat distribution, hormonal status, and muscle mass, to better clarify the gender-specific mechanisms linking BMI to ADL disability in older adults.

The current study has several notable strengths. First, we conducted the analyses by using a representative cohort of Chinese older adults with a long-term follow-up and a large sample size to ensure our findings are generalisable. Second, accurate measurement of height and weight using standardised protocols and appropriate categorisation of BMI according to the Chinese guideline of obesity increases the reliability of our results. Third, stepwise covariate adjustments and multiple subgroup and sensitivity analyses confirmed the robustness of our findings. Overall, our findings have significant public health implications. Screening for BMI should be incorporated into routine elderly assessments to facilitate early identification of individuals at elevated risk of ADL disability. While a BMI near 21 kg/m^2^ appears to correspond to the lowest risk, this cutoff requires further validation. Integrating BMI evaluation into elderly care systems and community health programmes could guide targeted interventions to maintain optimal BMI, thereby preserving physical function and reducing the burden of ADL disability.

This study also has several limitations. First, although we conducted sensitivity analyses by restricting the sample to participants with stable BMI values and used BMI measured at the second wave as the exposure variable in model construction, relying solely on baseline BMI still limits our ability to assess the impact of BMI changes over time on the risk of ADL disability. Second, the observational design restricts causal inference, and the underlying mechanism for the association among older adults remains unclear. Third, despite stepwise adjustment for important covariates, residual confounders may persist, potentially limiting our understanding of the association between BMI and ADL disability. Fourthly, while we performed subgroup analyses stratified by baseline urban-rural residence, we did not track changes in urban-rural distribution during follow-up. Given the potential differences in health care access, lifestyle, and environmental factors between urban and rural settings, these unmeasured changes may have influenced the observed associations and limited the generalisability of our findings. Finally, since the study included only Chinese older adults, the applicability of our results to other populations and geographic regions may be limited.

## CONCLUSIONS

In this nationwide cohort study, a nonlinear U-shaped relationship was observed between BMI and the risk of ADL disability among Chinese older adults, indicating that both high and low BMI are associated with increased ADL disability risk. This association was particularly robust among individuals aged 60–69 and females. These findings support the incorporation of BMI screening into routine geriatric assessments, with special emphasis on adults aged 60–69 and female populations. Tailored interventions, including weight management, nutritional counselling, and functional training, should be implemented according to age and gender to effectively prevent or delay the onset of ADL disability.

## Additional material


Online Supplementary Document
