# Supplementary material for: Association between body mass index and disability among older population in China: analysis of 2011–2020 data from the China Health and Retirement Longitudinal Study
Source: J Glob Health. 2025 Dec 5;15:04277. doi: 10.7189/jogh.15.04277 (PMC12679055; doi:10.7189/jogh.15.04277)
Supplement: Online Supplementary Document [file jogh-15-04277-s001.pdf]

Supplement to: Guo R, Zhang S, Fu T, Guan Y, Luo Y, Wu Y. Association between body mass index and disability among older population in China: analysis of 2011 – 2020 data from the China Health and Retirement Longitudinal Study. J Glob Health. 2025;15:04277.

Supplementary Method 1. The detailed description of the CHARLS 2

Supplementary Method 2. Specific items and definitions of covariates...................... 3

Table S1. The assignment methods of covariates 6

Table S2. Missing rates of data prior to application of multiple imputation 7

Table S3. Comparison of model performance between unadjusted and adjusted models in the primary analyses using the imputed dataset 8

Table S4. Inflection point and corresponding HRs for Models 1-4.............................. 9

Table S5. Cox regression model results (Model 4) for the association between categorical BMI and ADL disability in the subgroup analyses.................................... 10

Fig S1. Restricted cubic spline curves between continuous BMI and ADL disability for the subgroup analyses by age group 12

Fig S2. Restricted cubic spline curves between continuous BMI and ADL disability for the subgroup analyses by gender 13

Fig S3. Restricted cubic spline curves between continuous BMI and ADL disability for the subgroup analyses by marital status.. 14

Fig S4. Restricted cubic spline curves between continuous BMI and ADL disability for the subgroup analyses by education level.. 15

Fig S5. Restricted cubic spline curves between continuous BMI and ADL disability for the subgroup analyses by residence area 16

Table S6. Cox regression model results (Model4) for the association between continuous BMI and ADL disability in the sensitivity analyses 17

Table S7. Cox regression model results (Model 4) for the association between categorical BMI and ADL disability in the sensitivity analyses 18

Figure S6. Restricted cubic spline curves between continuous BMI and ADL disability for sensitivity analyses.. 20

Figure S7. Mechanisms underlying the association between high BMI and ADL disability... 22

References.. 23

Supplementary Method 1. The detailed description of the CHARLS

China Health and Retirement Longitudinal Study (CHARLS) is a national survey of Chinese residents aged 45 years and above. To ensure a representative sample, the CHARLS baseline survey conducted in 2011 covered 150 counties or districts and 450 villages or urban communities across 28 provinces by using multistage stratified probability-proportionate-to-size sampling. The CHARLS team obtained a full list of all residents in each region and randomly selected households to complete the survey. One resident aged 45 years or older was randomly chosen and their spouse was automatically included from each selected household. If there was no individual aged 45 years or older in the selected household, then the household was excluded. A total of 17708 individuals from 10257 households were successfully interviewed for the 2011 baseline survey.

Supplementary Method 2. Specific items and definitions of covariates

Sociodemographic characteristics, including age (continuous), gender (male or female) [1], education (illiterate, primary school or below, or secondary school or above), marital status (married or unmarried), residential area (urban or rural), and annual per-capita household expenditure (low, middle, or high level) [2] were collected using a predefined standard questionnaire.

Within the disease category, self-reported heart diseases, hypertension, diabetes or high blood sugar, arthritis, chronic lung diseases, stroke, and cancer were collected [3].

Functional impairments were assessed through self-reported vision [4] and hearing impairments [5] (yes or no), as well as objective measures of grip strength, lower-extremity function, and fall history [6]. Grip strength was measured twice in both hands, and the highest value from the dominant hand was used to determine low grip strength. Low grip strength was defined as <28 kg for men and <18 kg for women [7]. Lower-extremity function was evaluated using the Short Physical Performance Battery (SPPB), which includes balance, walking speed, and chair-stand tests [8]. For the balance test, participants were instructed to hold three progressively difficult stances: side-by-side, semi-tandem (heel beside the big toe), and full tandem (heel directly in front of the other foot), each for 10 seconds. Scores were assigned as follows: 1 point if participants could hold the side-by-side position for 10 seconds but not the semi-tandem position; 2 points if they held the semi-tandem position for 10 seconds but maintained the full tandem position for 2 seconds or less; 3 points if they held the full tandem position between 3 and 9 seconds; and 4 points if they maintained the full tandem position for the entire 10 seconds [9]. Walking speed test consisted of two timed 2.4-meter walks, with the faster

time used for scoring: >5.7 seconds (1 point), 4.1-5.6 seconds (2 points), 3.2-4.0seconds (3 points), and <3.1 seconds (4 points). For the chair-stand test, participants crossed their arms over their chest and first completed a single stand. If successful, they proceeded to perform five consecutive stands as quickly as possible. Scores were assigned based on completion time: >16.7 seconds (1 point), 13.7-16.6 seconds (2 points), 11.2-13.6 seconds (3 points), and <11.1 seconds (4 points). The total SPPB score, ranging from 3 to 12, was calculated by summing the three component scores and categorized as low (3-6), medium (7-9), and high (10-12) groups. Fall history was defined as having experienced one or more falls within the past two years.

Cognitive function was assessed using four tests: immediate and delayed word recall (each scored 0-10 points), time orientation (0-4 points), and serial 7's subtraction (0-5 points), yielding a total score of 0-29, with lower scores indicating worse cognition. Cognitive decline was defined as scoring at least 1.5 standard deviations (SDs) below the mean on two or more tests; others were considered cognitively normal [10]. Depression was evaluated using the 10-item Center for Epidemiologic Studies Depression Scale (CES-D-10) [11]. Participants reported the frequency of specific symptoms experienced during the past week across four response categories. Scores from the ten items were summed (0-30 points), with a total score≤10 indicating absence of depression, and scores >10 indicating presence of depression [12].

Regarding health behaviors, we collected smoking (never, former, or current smokers) and alcohol drinking (never, former, or current drinkers) [13]. Sleep quality was not assessed separately, as it was included within the CES-D-10 [14]. Physical activity

was excluded as a covariate due to a high proportion of missing data, which could compromise the sample size and the robustness of the analysis [15].

Under the social support domain, monthly social engagement (yes or no) was assessed [16]. Participants were asked whether they had engaged in any of the following activities in the past month: socializing with friends, playing games (e.g., Mahjong, chess, cards), attending community or sports clubs, participating in community organizations, or volunteering. Engagement in any of these activities qualified as having monthly social engagement; otherwise, participants were classified as not socially engaged.

Table S1. The assignment methods of covariates

Variables	Assignment methods
Age	ba002_1
Gender	Male: gender=1; Female: gender=2
Education	Illiterate: bd001=1;<br>Primary school or below: bd001=2/3/4;<br>Secondary school or above: bd001=5/6/7/8/9/10/11
Marital status	Married: be001=1/2/3; Unmarried: be001=4/5/6
Residence area	Urban residence: urban_nbs=1; Rural residence: urban_nbs=2
Annual per-capita household expenditure	The total annual expenditure of all household members, divided by the number of household members, expressed in local currencies.<br>Low level: bottom tertile;<br>Middle level: middle tertile;<br>High level: top tertile
Vision impairment	Yes: da033=4/5; No: da033=1/2/3
Hearing impairment	Yes: da039=4/5; No: da039=1/2/3
Hypertension	Yes: da007_1=1; No: da007_1=0
Diabetes or high blood sugar	Yes: da007_3=1; No: da007_3=0
Heart problems	Yes: da007_7=1; No: da007_7=0
Stroke	Yes: da007_8=1; No: da007_8=0
Cancer	Yes: da007_4=1; No: da007_4=0
Chronic lung diseases	Yes: da007_5=1; No: da007_5=0
Arthritis	Yes: da007_13=1; No: da007_13=0
Grip strength	if qc002=1, grip strength=max(qc004, qc006);<br>if qc002=2, grip strength=max(qc003, qc005);<br>if qc002=3, grip strength=max(qc004, qc006, qc003, qc005)
Smoking	Never smoker=1; Former smoker=2; Current smoker=3
Alcohol drinking	Never drinker=1; Former drinker=2; Current drinker=3
Cognitive Decline	Immediate word recall (0-10) + delayed word recall (0-10) + time orientation (0-4) + serial 7's (0-5)<br>Yes: performed 1.5 standard deviations below the mean of the score in two or three tests=1;<br>No: others=0
Depression	CES-D-10 scale (0-30)<br>Yes: CES-D-10 >10; No: CES-D-10≤10
Monthly social interactions	Yes: any of the selected social activities;<br>No: none of the selected social activities
SPPB level	Low level: 3≤SPPB score≤6;<br>Middle level: 7≤SPPB score≤9;<br>High level: 10≤SPPB score≤12

CES-D – Center for Epidemiologic Studies Depression Scale, SPPB – Short Physical Performance Battery.

Table S2. Missing rates of data prior to application of multiple imputation

**Variables**	**No. of missing values (%)**	**Variables**	**No. of missing values (%)**
Age	0 (0.00%)	**Gender**	0 (0.00%)
Marital status	3 (0.08%)	**Education**	3 (0.08%)
Annual per-capita household expenditure	754 (18.97%)	**Residence area**	0 (0.00%)
Vision impairment	70 (1.76%)	**Hearing impairment**	19 (0.48%)
Smoking	18 (0.45%)	**Alcohol drinking**	5 (0.13%)
Hypertension	13 (0.33%)	**Diabetes or high blood sugar**	25 (0.63%)
Stroke	6 (0.15%)	**Heart problems**	15 (0.38%)
Chronic lung diseases	11 (0.28%)	**Cancer**	20 (0.50%)
Arthritis	7 (0.17%)	**Fall history**	38 (0.96%)
Depression	184 (4.63%)	**Cognitive decline**	358 (9.01%)
SPPB level	446 (11.22%)	**Grip strength (kg)**	88 (2.21%)
Monthly social interaction	165 (4.15%)	**Low grip strength**	88 (2.21%)

SPPB – Short Physical Performance Battery.

Table S3. Comparison of model performance between unadjusted and adjusted models in the primary analyses using the imputed dataset

Model	AIC	Concordance index
Model 1*	31677.29	0.509
Model 1†	31408.57	0.617
Model 3‡	31290.61	0.646
Model 4 §	31114.68	0.678

AIC – Akaike Information Criterion.

*Unadjusted.

†Adjusted for age, gender, education, marital status, residence area, and annual per-capita household expenditure.

‡Adjusted for age, gender, education, marital status, residence area, annual per-capita household expenditure, hypertension, diabetes or high blood sugar, stroke, cancer, heart problems, chronic lung diseases, and arthritis.

§Adjusted for age, gender, education, marital status, residence area, annual per-capita household expenditure, hypertension, diabetes or high blood sugar, stroke, cancer, heart problems, chronic lung diseases, arthritis, smoking, drinking, low grip strength, vision impairment, hearing impairment, cognitive decline, depression, monthly social engagement, Short Physical Performance Battery level, and fall history.

Table S4. Inflection point and corresponding HRs for Models 1-4

Model	Inflection point	HR on the left side	HR on the right side
Model 1*	22.41	0.930 (0.889-0.973) *p*=0.001¶	1.027 (1.012-1.043) *p*=0.012¶
Model 1†	21.10	0.954 (0.907-1.004) *p*=0.071	1.024 (1.010-1.038) *p*<0.001¶
Model 3‡	21.54	0.974 (0.931-1.020) *p*=0.265	1.019 (1.003-1.034) *p*=0.019¶
Model 4§	20.96	0.976 (0.924-1.030) p=0.368	1.022 (1.008-1.038) *p*=0.003¶

HR – hazard ratio.
Data were presented as hazard ratios, 95% confidence intervals, and *p*.
*Unadjusted.
†Adjusted for age, gender, education, marital status, residence area, and annual per-capita household expenditure.
‡Adjusted for age, gender, education, marital status, residence area, annual per-capita household expenditure, hypertension, diabetes or high blood sugar, stroke, cancer, heart problems, chronic lung diseases, and arthritis.
§Adjusted for age, gender, education, marital status, residence area, annual per-capita household expenditure, hypertension, diabetes or high blood sugar, stroke, cancer, heart problems, chronic lung diseases, arthritis, smoking, drinking, low grip strength, vision impairment, hearing impairment, cognitive decline, depression, monthly social engagement, Short Physical Performance Battery level, and fall history.
¶Statistically significant (*p*<0.05).

Table S5. Cox regression model results (Model 4) for the association between categorical BMI and ADL disability in the subgroup analyses

**Subgroup analyses**	**Normal weight**	**Underweight**	**Overweight**	**Obesity**	***p*** **interaction**
Age group					all >0.05
60-69	Reference	1.110 (0.896, 1.375)<br>*p*=0.338	1.103 (0.962, 1.264)<br>*p*=0.161	1.396 (1.160, 1.680)<br>*p*<0.001*	
70-79	Reference	0.946 (0.742, 1.207)<br>*p*=0.657	1.048 (0.854, 1.287)<br>*p*=0.654	1.167 (0.849, 1.604)<br>*p*=0.342*	
80-89	Reference	0.849 (0.503, 1.433)<br>*p*=0.540	0.991 (0.571, 1.718)<br>*p*=0.973	1.488 (0.689, 3.211)<br>*p*=0.312*	
Gender					all >0.05
Male	Reference	1.154 (0.920, 1.449)<br>*p*=0.216	0.995 (0.836, 1.184)<br>*p*=0.956	1.371 (1.052, 1.787)<br>*p*=0.019*	
Female	Reference	0.970 (0.790, 1.193)<br>*p*=0.775	1.148 (0.994, 1.327)<br>*p*=0.061	1.374 (1.134, 1.665)<br>*p*=0.001*	
Marital status					all >0.05
Married	Reference	0.997 (0.834, 1.191)<br>*p*=0.970	0.028 (0.909, 1.163)<br>*p*=0.658	1.331 (1.118, 1.585)<br>*p*=0.001*	
Unmarried	Reference	1.094 (0.812, 1.474)<br>*p*=0.553	1.303 (1.009, 1.683)<br>*p*=0.043*	1.444 (1.023, 2.037)<br>*p*=0.037*	
Education level					all >0.05
Illiterate	Reference	0.980 (0.786, 1.221)<br>*p*=0.854	0.996 (0.831, 1.194)<br>*p*=0.963	1.374 (1.082, 1.744)<br>*p*=0.009*	
Primary school or below	Reference	1.065 (0.846, 1.342)<br>*p*=0.590	1.139 (0.970, 1.338)<br>0.113	1.341 (1.055, 1.705)<br>*p*=0.017*	
Secondary school or above	Reference	1.097 (0.622, 1.933)<br>*p*=0.750	1.084 (0.803, 1.463)<br>*p*=0.599	1.528 (1.012, 2.308)<br>*p*=0.044*	
Residence area					all >0.05

Urban area	Reference	0.936 (0.680, 1.289) *p*=0.686	1.078 (0.893, 1.300) *p*=0.434	1.453 (1.152, 1.832) *p*=0.002*	
Rural area	Reference	1.047 (0.880, 1.246) *p*=0.603	1.100 (0.958, 1.262) *p*=0.177	1.276 (1.029, 1.584) *p*=0.027*	

BMI – body mass index, ADL – activities of daily living.

Data were presented as hazard ratios, 95% confidence intervals, and *p*.

*Statistically significant ($p<0.05$).

Association between BMI and ADL disability

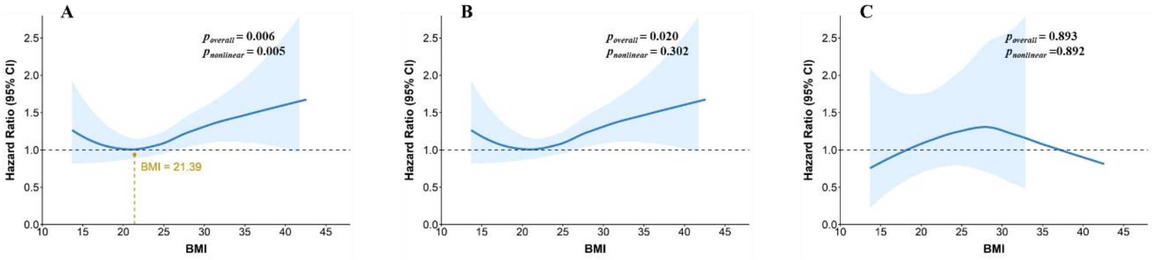

Fig S1. Restricted cubic spline curves between continuous BMI and ADL disability for the subgroup analyses by age group. **Panel A.** Aged 60-69 years old. **Panel B.** Aged 70-79 years old. **Panel C.** Aged 80 or above. BMI – body mass index. ADL – activities of daily living.

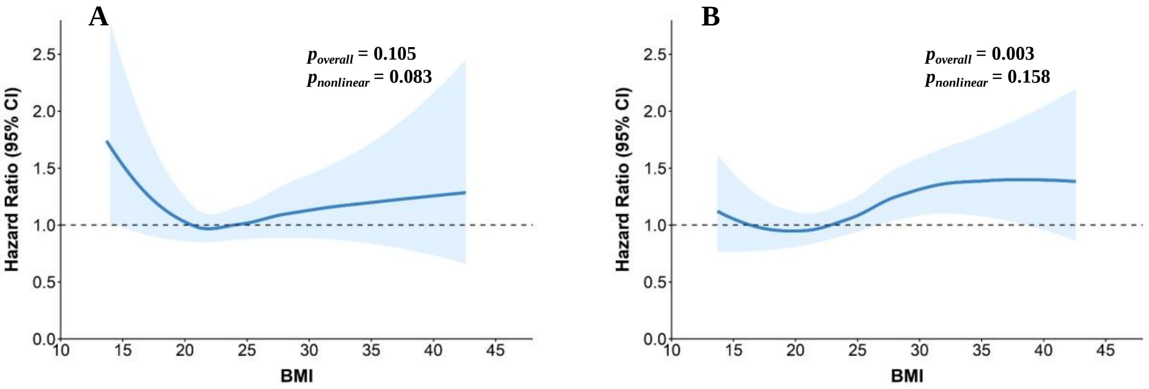

Fig S2. Restricted cubic spline curves between continuous BMI and ADL disability for the subgroup analyses by gender. **Panel A.** Male. **Panel B.** Female. BMI – body mass index. ADL – activities of daily living.

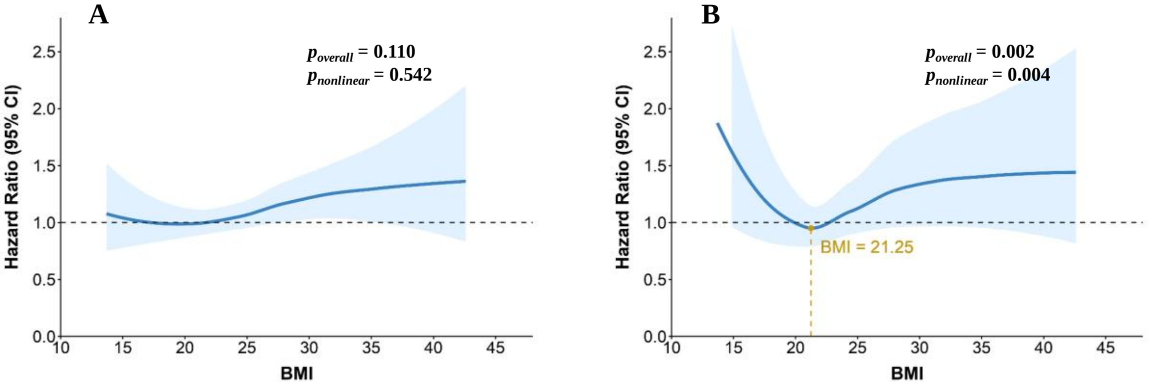

Fig S3. Restricted cubic spline curves between continuous BMI and ADL disability for the subgroup analyses by marital status. **Panel A.** Married individuals. **Panel B.** Unmarried individuals. BMI – body mass index. ADL – activities of daily living.

Association between BMI and ADL disability

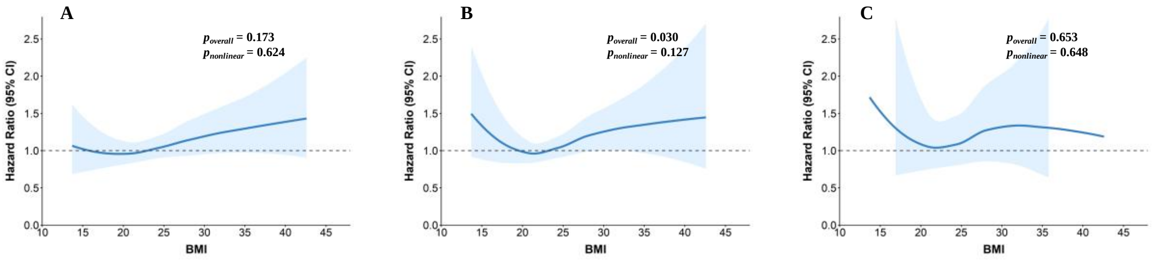

Fig S4. Restricted cubic spline curves between continuous BMI and ADL disability for the subgroup analyses by education level. **Panel A.** Illiterate group. **Panel B.** Primary school or below. **Panel C.** Secondary school or above. BMI – body mass index. ADL – activities of daily living.

Association between BMI and ADL disability

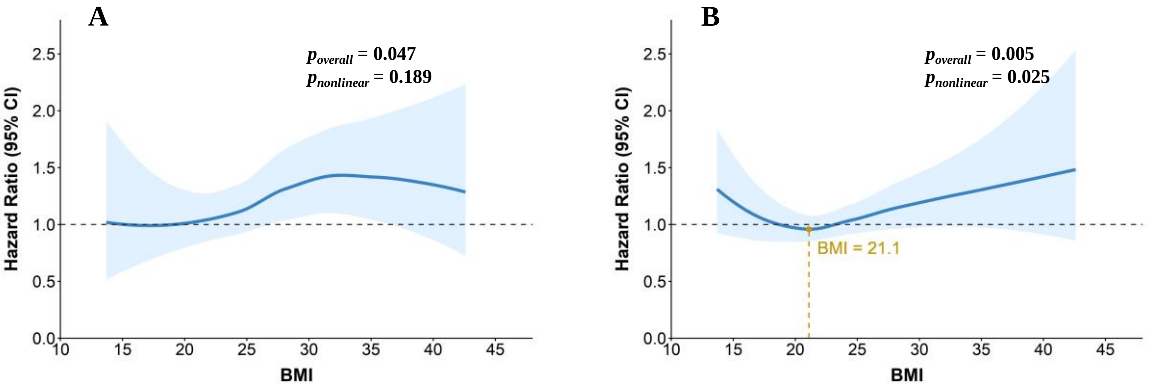

Fig S5. Restricted cubic spline curves between continuous BMI and ADL disability for the subgroup analyses by residence area. **Panel A.** Urban residents. **Panel B.** Rural residents.

Association between BMI and ADL disability

Table S6. Cox regression model results (Model4) for the association between continuous BMI and ADL disability in the sensitivity analyses

Sensitivity analyses	No. of events	HR (95%CI)	*p*
By conducting a lagged panel analysis	2003/3975	1.029 (1.014, 1.044)	<0.001*
By excluding participants who died at follow-up	1599/3339	1.021 (1.009, 1.034)	<0.001*
By using BMI measured at the first follow-up for model construction	1537/2963	1.007 (1.001, 1.013)	0.033*
By excluding participants with unstable BMI between baseline and the first follow-up	927/1885	1.016 (1.001, 1.032)	0.041*
By excluding participants who developed ADL disability at the first follow-up	1700/3538	1.020 (1.008, 1.033)	0.002*
By excluding participants with missing covariates	1362/2767	1.018 (1.003, 1.032)	0.015*
By excluding participants with cognitive decline	1639/3398	1.017 (1.004, 1.031)	0.009*
By excluding participants with depression	1249/2838	1.018 (1.005, 1.032)	0.009*
By excluding participants with low grip strength	1584/3288	1.021 (1.009, 1.034)	<0.001*
By excluding participants with hypertension	1352/2838	1.026 (1.010, 1.041)	<0.001*
By excluding participants with diabetes or high blood sugar	1855/3717	1.019 (1.007, 1.031)	0.002*
By excluding participants with stroke	1944/3888	1.020 (1.009, 1.032)	<0.001*
By excluding participants with heart problems	1686/3423	1.018 (1.006, 1.031)	0.004*
By excluding participants with cancer	1984/3931	1.019 (1.007, 1.030)	0.001*
By excluding participants with arthritis	1215/2636	1.016 (1.002, 1.031)	0.031*
By excluding participants with fall history	1607/3324	1.020 (1.008, 1.033)	0.002*
By excluding participants with vision impairment	1343/2904	1.019 (1.005, 1.033)	0.006*
By excluding participants with hearing impairment	1578/3256	1.022 (1.010, 1.035)	<0.001*

BMI – body mass index, ADL – activities of daily living, HR – hazard ratio, CI – confidence interval.
*Statistically significant (p<0.05).

Table S7. Cox regression model results (Model 4) for the association between categorical BMI and ADL disability in the sensitivity analyses

Subgroup analyses	Normal weight	Underweight	Overweight	Obesity	$p_{interaction}$
By conducting a lagged panel analysis	Reference	1.025 (0.838, 1.254) *p*=0.810	1.122 (0.980, 1.284) *p*=0.096	1.442 (1.199, 1.735) *p*<0.001*	all >0.05
By excluding participants who died at follow-up	Reference	1.110 (0.933, 1.321) *p*=0.240	1.110 (0.983, 1.254) *p*=0.094	1.411 (1.193, 1.669) *p*<0.001*	all >0.05
By using BMI measured at the first follow-up for model construction	Reference	1.080 (0.887, 1.315) *p*=0.443	1.079 (0.926, 1.259) *p*=0.329	1.299 (1.038, 1.626) *p*=0.022*	all >0.05
By excluding participants with unstable BMI between baseline and the first follow-up	Reference	0.954 (0.748, 1.217) *p*=0.704	1.001 (0.852, 1.176) *p*=0.989	1.287 (0.016, 1.630) *p*=0.036*	all >0.05
By excluding participants who developed ADL disability at the first follow-up	Reference	1.028 (0.871, 1.214) *p*=0.743	1.099 (0.976, 1.237) *p*=0.118	1.370 (1.159, 1.619) *p*<0.001*	all >0.05
By excluding participants with missing covariates	Reference	0.981 (0.812, 1.184) *p*=0.839	1.070 (0.935, 1.225) *p*=0.324	1.309 (1.087, 1.577) *p*=0.005*	all >0.05
By excluding participants with cognitive decline	Reference	1.023 (0.858, 1.220) *p*=0.802	1.091 (0.968, 1.231) *p*=0.154	1.297 (1.098, 1.533) *p*=0.002*	all >0.05
By excluding participants with depression	Reference	1.134 (0.926, 1.389) *p*=0.223	1.160 (1.011, 1.330) *p*=0.034*	1.382 (1.142, 1.671) *p*<0.001*	all >0.05
By excluding participants with low grip strength	Reference	1.032 (0.862, 1.235) *p*=0.731	1.093 (0.968, 1.234) *p*=0.151	1.378 (1.162, 1.634) *p*<0.001*	all >0.05
By excluding participants with hypertension	Reference	1.075 (0.910, 1.269) *p*=0.394	1.139 (0.991, 1.309) *p*=0.067	1.494 (1.205, 1.852) *p*<0.001*	all >0.05
By excluding participants with diabetes or high blood sugar	Reference	1.046 (0.896, 1.220) *p*=0.570	1.125 (1.003, 1.262) *p*=0.044*	1.349 (1.144, 1.592) *p*<0.001*	all >0.05
By excluding participants with stroke	Reference	1.025 (0.879, 1.195) *p*=0.755	1.066 (0.952, 1.192) *p*=0.269	1.401 (1.197, 1.639) *p*<0.001*	all >0.05

Association between BMI and ADL disability

By excluding participants with heart problems	Reference	1.067 (0.909, 1.253) *p*=0.430	1.094 (0.969, 1.235) *p*=0.147	1.393 (1.167, 1.662) *p*<0.001*	all >0.05
By excluding participants with cancer	Reference	1.023 (0.878, 1.191) *p*=0.775	1.071 (0.959, 1.197) *p*=0.225	1.346 (1.152, 1.573) *p*<0.001*	all >0.05
By excluding participants with arthritis	Reference	1.120 (0.926, 1.354) *p*=0.244	1.056 (0.916, 1.217) *p*=0.452	1.430 (1.166, 1.752) *p*<0.001*	all >0.05
By excluding participants with fall history	Reference	1.065 (0.898, 1.261) *p*=0.470	1.166 (1.033, 1.318) *p*=0.013*	1.359 (1.142, 1.617) *p*<0.001*	all >0.05
By excluding participants with vision impairment	Reference	0.959 (0.795, 1.158) *p*=0.665	1.063 (0.929, 1.216) *p*=0.375	1.332 (1.104, 1.608) *p*=0.003*	all >0.05
By excluding participants with hearing impairment	Reference	1.108 (0.932, 1.318) *p*=0.247	1.161 (1.026, 1.313) *p*=0.018*	1.433 (1.207, 1.700) *p*<0.001*	all >0.05

BMI – body mass index, ADL – activities of daily living.
Data were presented as hazard ratios, 95% confidence intervals, and *p*.
*Statistically significant (*p*<0.05)..

Association between BMI and ADL disability

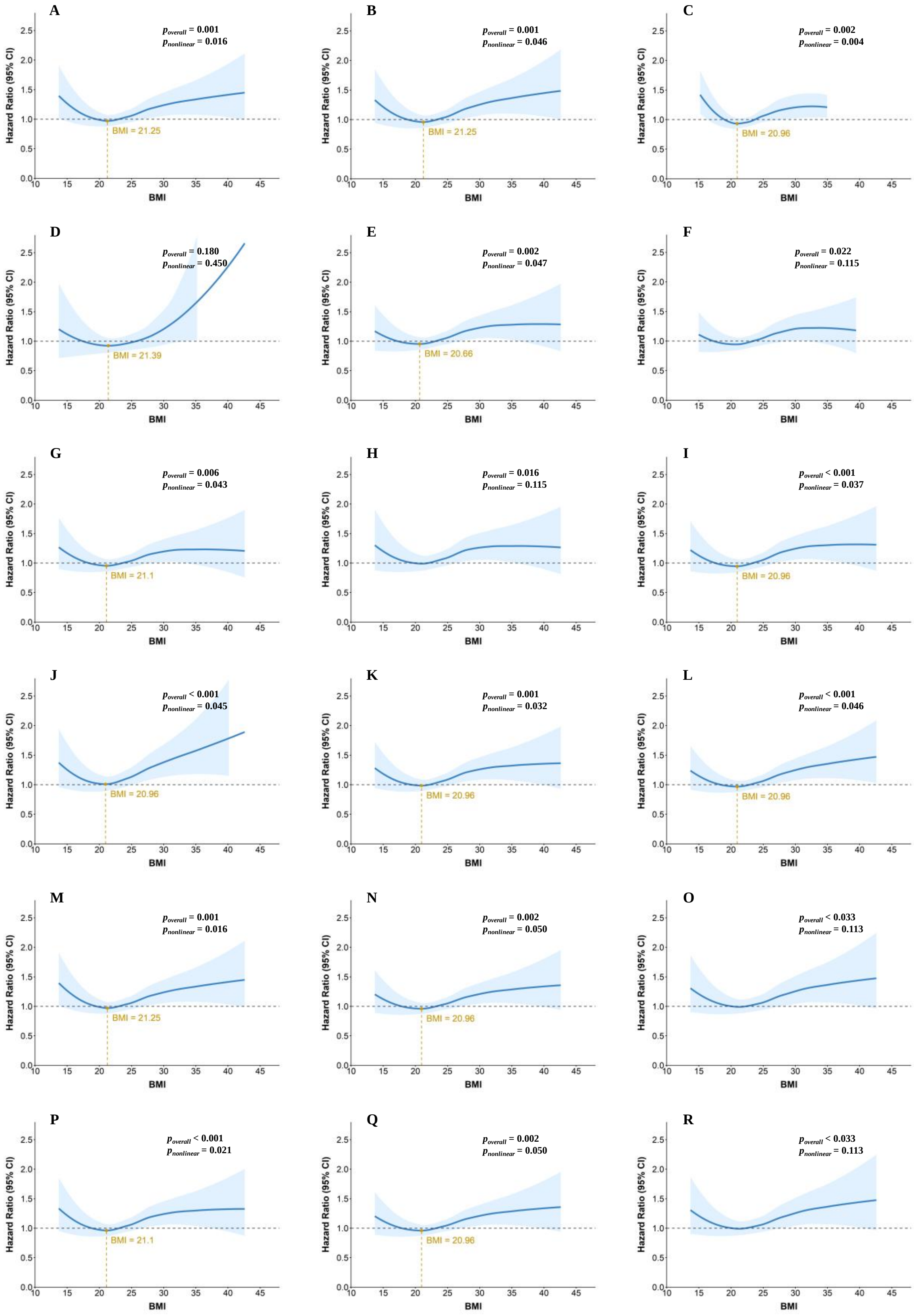

Figure S6. Restricted cubic spline curves between continuous BMI and ADL disability for sensitivity analyses. **Panel A.** By conducting a lagged panel analysis. **Panel B.** By excluding participants who died at follow-up. **Panel C.** By using BMI measured at the first follow-up for model construction. **Panel D.** By excluding participants with unstable

Association between BMI and ADL disability

BMI between baseline and the first follow-up. **Panel E.** By excluding participants who developed ADL disability at the first follow-up. **Panel F.** By excluding participants with missing covariates. **Panel G.** By excluding participants with cognitive decline. **Panel H.** By excluding participants with depression. **Panel I.** By excluding participants with low grip strength. **Panel J.** By excluding participants with hypertension. **Panel K.** By excluding participants with diabetes or high blood sugar. **Panel L.** By excluding participants with stroke. **Panel M.** By excluding participants with heart problems. **Panel N.** By excluding participants with cancer. **Panel O.** By excluding participants with arthritis. **Panel P.** By excluding participants with fall history. **Panel Q.** By excluding participants with vision impairment. **Panel R.** By excluding participants with hearing impairment. BMI – body mass index. ADL – activities of daily living.

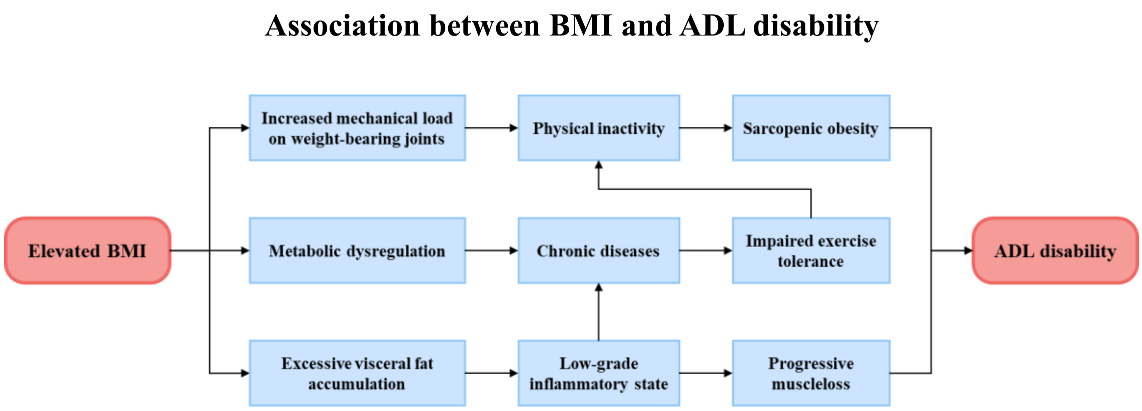

Figure S7. Mechanisms underlying the association between high BMI and ADL disability. BMI – body mass index. ADL – activities of daily living.

References

[1] Hao X, Zhang H, Zhao X, Peng X, Li K. Risk factors for hospitalization-associated disability among older patients: A systematic review and meta-analysis. Ageing Res Rev. 2024 Nov;101:102516.

[2] Liu H, Wang M. Socioeconomic status and ADL disability of the older adults: Cumulative health effects, social outcomes and impact mechanisms. PLoS One. 2022 Feb 10;17(2):e0262808.

[3] Botoseneanu A, Markwardt S, Quiñones AR. Multimorbidity and Functional Disability among Older Adults: The Role of Inflammation and Glycemic Status - An Observational Longitudinal Study. Gerontology. 2023;69(7):826-838.

[4] Zaninotto P, Maharani A, Di Gessa G. Vision and Hearing Difficulties and Life Expectancy Without ADL/IADL Limitations: Evidence From the English Longitudinal Study of Ageing and the Health and Retirement Study. J Gerontol A Biol Sci Med Sci. 2024 Feb 1;79(2):glad136.

[5] Lin TC, Yen M, Liao YC. Hearing loss is a risk factor of disability in older adults: A systematic review. Arch Gerontol Geriatr. 2019 Nov-Dec;85:103907.

[6] Bally ELS, Ye L, van Grieken A, Tan SS, Mattace-Raso F, Procaccini E, Alhambra-Borrás T, Raat H. Factors associated with falls among hospitalized and community-dwelling older adults: the APPCARE study. Front Public Health. 2023 Jun 29;11:1180914.

[7] Huang YC, Dong Y, Tang CM, Shi Y, Pang J. Mortality and disability risk among older adults unable to complete grip strength and physical performance tests: a population-based cohort study from China. BMC Public Health. 2024 Mar 13;24(1):797.

[8] Guralnik JM, Ferrucci L, Simonsick EM, Salive ME, Wallace RB. Lower-extremity function in persons over the age of 70 years as a predictor of subsequent disability. N Engl J Med. 1995 Mar 2;332(9):556-61.

[9] Braun T, Thiel C, Peter RS, Bahns C, Büchele G, Rapp K, Becker C, Grüneberg C. Association of clinical outcome assessments of mobility capacity and incident disability in community-dwelling older adults - a systematic review and meta-analysis. Ageing Res Rev. 2022 Nov;81:101704.

[10] Tang KF, Teh PL, Lee SWH. Cognitive Frailty and Functional Disability Among Community-Dwelling Older Adults: A Systematic Review. Innov Aging. 2023 Jan 23;7(1):igad005.

[11] Peng S, Wang S, Feng XL. Multimorbidity, depressive symptoms and disability in activities of daily living amongst middle-aged and older Chinese: Evidence from the China Health and Retirement Longitudinal Study. J Affect Disord. 2021 Dec 1;295:703-710.

[12] Ning H, Du Y, Zhao Y, Liu Q, Li X, Zhang H, Jiang D, Feng H. Longitudinal impact of metabolic syndrome and depressive symptoms on subsequent functional disability among middle-aged and older adults in China. J Affect Disord. 2022 Jan 1;296:216-223.

[13] Andresen EM, Malmgren JA, Carter WB, Patrick DL. Screening for depression in well older adults: evaluation of a short form of the CES-D (Center for Epidemiologic

Studies Depression Scale). Am J Prev Med. 1994 Mar-Apr;10(2):77-84.
[14] Zhou J, Xu Y, Yang D, Zhou Q, Ding S, Pan H. Risk prediction models for disability in older adults: a systematic review and critical appraisal. BMC Geriatr. 2024 Oct 2;24(1):806.
[15] Luo M, Dong Y, Fan B, Zhang X, Liu H, Liang C, Rong H, Fei Y. Sleep Duration and Functional Disability Among Chinese Older Adults: Cross-Sectional Study. JMIR Aging. 2024 Jun 10;7:e53548.
[16] Yu YY, Lai HY, Loh CH, Chen LK, Hsiao FY. Multi-trajectories of physical activity and health in aging: A 20-year nationwide population-based longitudinal study. Arch Gerontol Geriatr. 2025 Aug;135:105878.
